# Treatment patterns in a real-world cohort of patients with Wilson disease in the United States

**DOI:** 10.3389/fgstr.2024.1363130

**Published:** 2024-05-24

**Authors:** Valentina Medici, Nehemiah Kebede, Jennifer Stephens, Mary Kunjappu, John M. Vierling

**Affiliations:** ^1^ Department of Internal Medicine, Division of Gastroenterology and Hepatology, University of California (UC) Davis School of Medicine, Sacramento, CA, United States; ^2^ Evidence & Access, OPEN Health, Bethesda, MD, United States; ^3^ Alexion, AstraZeneca Rare Disease, Boston, MA, United States; ^4^ Section of Gastroenterology and Hepatology, Baylor College of Medicine, Houston, TX, United States

**Keywords:** chelating agents, copper, penicillamine, trientine, zinc

## Abstract

**Background:**

Wilson disease (WD) is a rare and potentially fatal genetic disorder caused by accumulation of toxic levels of copper. Current treatments include chelating agents and/or zinc. We characterized real-world US treatment patterns in patients with WD.

**Methods:**

This retrospective, observational medical chart review utilized deidentified clinical data, including treatment patterns, abstracted from patient medical charts between 01/2012 and 06/2017. Line of therapy was assessed based on disease presentation and aggregated. Index treatment was defined as the first line of therapy, followed by second line of therapy and third line of therapy. Results were summarized using descriptive statistics.

**Results:**

A total of 225 patients were included (mean [SD] age at diagnosis: 24.7 [9.8] years). Initial disease presentation was both neurologic/psychiatric and hepatic in 52.9%, followed by neurologic/psychiatric (20.0%), hepatic (16.9%), and asymptomatic (10.2%). Median (first and third quartiles) duration of follow-up from diagnosis was 39.5 (33.8–60.4) months. The most common first line of therapy was penicillamine monotherapy in 45.5%, followed by trientine monotherapy (26.1%) and chelator/zinc combination therapy (21.2%). A total of 167/222 (75.2%) patients remained on first line of therapy during the follow-up period. Of the 13.5% who switched to second line of therapy, most changed to trientine monotherapy (53.3%). All those who switched to third line of therapy transitioned to zinc monotherapy (100.0%). Unexpectedly, 11.3% discontinued first line of therapy without transitioning to a subsequent therapy. The primary rationale for index monotherapy selection was improved efficacy (61.6%). Most discontinuations were due to side effects/tolerability (40.8%). Treatment patterns varied by initial disease presentation, practice setting, physician specialty, and geographic location.

**Conclusion:**

These results demonstrate a lack of consensus in the US regarding first-line treatment for patients with WD. Evidence-based treatment pathways informed by high-quality clinical trials for improved health outcomes are needed.

## Introduction

Wilson disease (WD) is an inherited genetic disorder caused by mutations in *ATP7B*, the hepatobiliary copper-transporting gene (1). The disease is rare, affecting approximately 1 in 30,000 to 50,000 individuals in the United States, although estimates vary (2). People with WD accumulate copper because of a genetic inability to excrete copper, resulting in harmful levels of copper deposition within the tissues and organs, particularly the brain, corneae, and liver (3).

Although organ damage caused by copper can begin in childhood, the clinical signs and symptoms often do not appear until the second or third decades of life (4). In a cohort of 62 patients participating in a US and European WD registry, the median age at diagnosis was 19 years (interquartile range, 11–25 years) (5). Unfortunately, the average time from onset of symptoms or signs of WD to diagnosis is typically more than 2 years (6–11). Delayed diagnosis or misdiagnosis is common, as WD signs and symptoms are heterogeneous, its phenotypic manifestations differ among patients, and clinicians often attribute signs/symptoms to other diseases (6, 10–14). Additionally, the fact that no single diagnostic test can conclusively establish or exclude a WD diagnosis requires clinicians to order several sequential diagnostic tests (1).

Patients with WD may be asymptomatic or exhibit symptoms or signs of hepatic and neurologic/psychiatric dysfunction in varying combinations (1, 12). Hepatic disease presentation has been reported in 15%–83% of patients (7, 15–21), manifesting variably as acute or chronic hepatitis, cirrhosis, or acute liver failure (1). Neurologic disease presentation has been reported in 20%–82% of patients (7, 15–21), manifesting as dysarthria, dystonia, tremor, and parkinsonism (13). Kayser-Fleischer rings (copper deposits within the corneae) have been identified in patients with neurologic symptoms or signs but are detectable in only a variable proportion of patients with hepatic disease (1, 15, 22, 23). Psychiatric presentation of WD has been reported in 2%–23% of patients (15, 17), primarily manifesting as depression, incongruous behavior, cognitive impairment, and irritability (24). Finally, asymptomatic disease with no organ damage has been identified in 3%–29% of patients with WD (15, 17–19).

WD is progressive and fatal if untreated (22). Historically, penicillamine has been the mainstay of WD treatment, because it was the first oral copper chelating agent recommended for WD treatment used extensively worldwide (3, 25). Subsequently, trientine was approved as an alternative copper chelating agent for WD treatment (25). Later, zinc gluconate, proven to reduce intestinal absorption of dietary copper, was introduced for use as monotherapy or in combination with a chelating agent (25, 26).

Three treatment guidelines for the treatment of WD have been published (3, 22, 27). WD guidance from the American Association for the Study of Liver Diseases (AASLD, 2022) is the most recent (26). The guidelines of the European Association for the Study of the Liver (EASL) were published in 2012 (22, 26), and those of the European Society for Paediatric Gastroenterology, Hepatology and Nutrition (ESPGHAN) were published in 2018 (27). All guidelines recommend a low-copper diet, especially during the first year of treatment (22, 26, 27). These guidelines agree overall on the treatment of liver disease but agree to a lesser extent on the treatment of neurologic and psychiatric manifestations of WD (14). All guidelines support initial first line of therapy of symptomatic patients with copper chelation using penicillamine or trientine. Two guidelines (AASLD and ESPGHAN) propose initial combination first line of therapy with a chelator plus zinc for patients with decompensated cirrhosis (22, 26, 27). EASL uniquely proposed zinc as first line of therapy in patients with neurologic disease (22). For asymptomatic patients without evidence of organ damage (or presymptomatic disease), both AASLD and EASL recommend first line of therapy with a chelator or zinc, while ESPGHAN favors zinc monotherapy.

In rare diseases like WD, real-world data are germane to understanding clinical characteristics and treatment outcomes and identifying unmet needs (28). The aim of the current study was to assemble real-world data from US physicians who treat patients with WD to assess 3 key questions. First, what is the spectrum of WD clinical presentations? Second, what were the rationales for first-line and subsequent therapies among physicians of different specialties, types of practices, and geographic sites of practice? Third, what proportion of physicians used published WD guidelines in management of their patients with WD?

## Methods

### Study design and objectives

This retrospective, observational medical chart review study used deidentified clinical data abstracted directly from medical records of US patients diagnosed with WD. The study’s objective was to describe patient treatment patterns in real-world clinical practice. In the current study, data were abstracted for patients identified by their treating physicians as having an initial WD diagnosis between January 1, 2012, and June 30, 2017. For analysis, patients were required to have ≥12 months of clinical data available both before and after their initial WD diagnosis. Patient-level clinical data were collected from the 12-month period prior to diagnosis through their most recent visit or death. The index treatment was defined as first line of therapy, which could be either monotherapy or combination therapy.

Ethics committee approval for this study was obtained from the New England Institutional Review Board (Needham, MA) on January 16, 2020.

### Data source and study cohort

A geographically dispersed sample of physicians treating patients with WD, comprising neurologists, hepatologists, and gastroenterologists, were selected from national physician databases for each specialty and recruited by email, telephone, and/or fax. Physicians were interviewed prior to their participation using a customized questionnaire to confirm their experience in managing WD. Eligible physicians had to have managed and/or treated at least 5 patients with WD between January 1, 2012, and June 30, 2017, who had data for ≥12 months prior to and after diagnosis, to be willing to collect all data of interest, and to agree with study requirements regarding validation of data and resolution of data queries. Physicians who qualified were responsible for identifying their eligible patients, extracting patient data, and completing case report forms. Before study initiation, all data collection forms were pretested with a minimum of 2–3 WD-treating physicians to ensure reliability and validity.

Eligible patients were ≥3 years of age when initially diagnosed with WD between January 1, 2012, and June 30, 2017. Each had a complete medical history available from diagnosis through the most recent visit or death, whichever occurred first, and were not currently enrolled in any WD-related clinical trials.

Key demographic physician data included years in practice, medical specialty, practice setting, type of hospital, geographic location, and use of WD treatment guidelines in their practice. Key data extracted from patient charts included patient demographic and clinical/disease characteristics (e.g., disease presentation, symptoms, and signs). Disease presentation at diagnosis was categorized as: (1) neurologic/psychiatric, (2) hepatic, (3) neurologic/psychiatric and hepatic, or (4) asymptomatic. For patients treated with an index therapy of penicillamine, trientine, or zinc monotherapy (or penicillamine/trientine in combination with zinc), additional data were extracted regarding treatment history, duration of treatment, and the rationale for selection of monotherapy as first line of therapy. Treatment duration was defined as time from treatment initiation to earliest discontinuation of first line of therapy or end of follow-up/death. The rationales for treatment selection/discontinuation were selected by physicians from a list of predefined options, and more than 1 response could be selected.

### Statistical analysis

Descriptive statistics were used to summarize patient demographics, clinical characteristics, and treatment patterns. Patient data were deidentified and reported in aggregate. Categorical variables of interest were summarized using the number and percentage of patients in each category. Continuous variables were summarized using mean, standard deviation (SD), median, first and third quartiles (Q1, Q3), and minimum and maximum values. Patient treatment patterns were analyzed overall and were also stratified according to initial disease presentation, practice setting, physician type, and geographic location. Queries regarding missing or incomplete data were resolved directly with participating physicians.

## Results

### Demographic and clinical characteristics

A total of 44 physicians participated in the study and provided data on 225 patients. Among the 225 patients, 222 received index treatment (monotherapy, n=172; combination therapy, n=50), and 3 received no treatment. Median (Q1–Q3) duration of follow-up from diagnosis for all patients was 39.5 (33.8–60.4) months.

**Table 1** summarizes the demographic and practice features of study physicians. Over one-third of physicians had been in practice for ≤10 years (16/44 [36.3%]) and, at the time of the study, were working in urban (26/44 [59.1%]), academic (23/44 [52.3%]), and/or university-based hospital (25/44 [56.8%]) settings. The largest proportion of physicians were gastroenterologists and hepatologists, accounting together for more than 75% of physicians (19/44 [43.2%] and 15/44 [34.1%], respectively), followed by neurologists (10/44 [22.7%]). A total of 18/44 (40.9%) physicians reported not using WD treatment guidelines in practice. On average, physicians reported prescribing dietary restrictions to only 57.7% of their patients (data not shown).

**Table 1:** Physician demographic and practice characteristics.

Variable	Statistic/Category	US (N=44)
Time in practice, y	2–5	2 (4.5)
	6–10	14 (31.8)
	11–15	9 (20.5)
	16–20	8 (18.2)
	21–30	11 (25.0)
Primary medical specialty	Neurology	10 (22.7)
	Hepatology	15 (34.1)
	Gastroenterology	19 (43.2)
Practice setting	Academic institution	23 (52.3)
	Nonacademic institution	7 (15.9)
	Private practice	14 (31.8)
Type of hospital	University-based	25 (56.8)
	General	5 (11.4)
	Regional	6 (13.6)
	Community	18 (40.9)
Location	Urban	26 (59.1)
	Rural	3 (6.8)
	Suburban	15 (34.1)
WD treatment guidelines used in practice	Yes	26 (59.1)
	No	18 (40.9)
Patients treated for WD by the physician	n	44
	Mean (SD)	11.3 (15.7)
	Median (Q1–Q3)	8.0 (3.0–13.0)

Values are n (%) unless otherwise specified.

Q, quartile; SD, standard deviation; WD, Wilson disease.

**Table 2** presents demographic and clinical characteristics for all patients and those who initiated index monotherapy. Mean (SD) patient age was 29.1 (9.7) years (range, 12–68 years) at the time of the study, 23.2 (8.9) years at onset of symptoms, and 24.7 (9.8) years at WD diagnosis. Most patients (147/225 [65.3%]) were male and had a Charlson Comorbidity Index of 0 (177/225 [78.7%]) at diagnosis (29). Symptoms at diagnosis were reported as hepatic in 157/225 (69.8%), as neurologic in 135/225 (60.0%), as psychiatric in 120/225 (53.3%), and as other in 90/225 (40.0%) patients. The most common disease presentations at WD diagnosis were a combination of both neurologic/psychiatric and hepatic (119/225 [52.9%]) dysfunction, followed by neurologic/psychiatric (45/225 [20.0%]), hepatic (38/225 [16.9%]), and asymptomatic (23/225 [10.2%]). Of these 225 patients, 18 (8.0%) had a liver transplant during the follow up period and 10 (4.4%) had died during the follow up period; 4 due to complications of their WD.

**Table 2:** Patient demographic and clinical characteristics.

Variable	Statistic/Category	All patients (N=225)	Index monotherapy (n=172)		
			Penicillamine (n=101)	Trientine (n=58)	Zinc (n=13)
Current age, mean (SD), years		29.1 (9.7)	32.2 (11.3)	24.4 (6.2)	27.7 (7.9)
Age at diagnosis, mean (SD), years		24.7 (9.8)	28.1 (11.4)	19.7 (6.5)	23.8 (8.0)
Age at first symptom, years	Mean (SD)	23.2 (8.9)	25.3 (10.9)	19.3 (6.6)	24.2 (1.3)
	Median (Q1–Q3)	22.0 (16.0–27.0)	23.0 (17.0–29.0)	17.0 (15.0–23.0)	24.0 (23.0–25.0)
	Range	8.0–63.0	12.0–63.0	9.0–36.0	23.0–26.0
Sex	Male	147 (65.3)	71 (70.3)	35 (60.3)	6 (46.2)
	Female	78 (34.7)	30 (29.7)	23 (39.7)	7 (53.8)
Race	White	199 (88.4)	87 (86.1)	54 (93.1)	12 (92.3)
	Black/African American	13 (5.8)	10 (9.9)	0 (0.0)	0 (0.0)
	Asian	9 (4.0)	1 (1.0)	4 (6.9)	1 (7.7)
	Native American/Alaska native	2 (0.9)	1 (1.0)	0 (0.0)	0 (0.0)
	Multiracial/Unknown	2 (0.9)	2 (2.0)	0 (0.0)	0 (0.0)
Ethnicity	Hispanic or Latino	17 (7.6)	6 (5.9)	6 (10.3)	0 (0.0)
	Not Hispanic or Latino	181 (80.4)	77 (76.2)	47 (81.0)	12 (92.3)
	Unknown	27 (12.0)	18 (17.8)	5 (8.6)	1 (7.7)
Family history of WD	Yes	51 (22.7)	17 (16.8)	21 (36.2)	2 (15.4)
Primary major medical insurance	Medicare	2 (0.9)	2 (2.0)	0 (0.0)	0 (0.0)
	Medicaid	48 (21.3)	15 (14.9)	13 (22.4)	3 (23.1)
	Commercial	174 (77.3)	84 (83.2)	44 (75.9)	10 (76.9)
	Cash/Uninsured	1 (0.4)	0 (0.0)	1 (1.7)	0 (0.0)
Primary pharmacy/drug insurance	Medicare	3 (1.3)	3 (3.0)	0 (0.0)	0 (0.0)
	Medicaid	47 (20.9)	14 (13.9)	13 (22.4)	3 (23.1)
	Commercial	174 (77.3)	84 (83.2)	44 (75.9)	10 (76.9)
	Cash/Uninsured	1 (0.4)	0 (0.0)	1 (1.7)	0 (0.0)
CCI at diagnosis	0	177 (78.7)	79 (78.2)	49 (84.5)	12 (92.3)
	1	27 (12.0)	10 (9.9)	5 (8.6)	0 (0.0)
	≥2	21 (9.3)	12 (11.9)	4 (6.9)	1 (7.7)
Symptoms at WD diagnosis	Neurologic	135 (60.0)	56 (55.4)	37 (63.8)	9 (69.2)
	Psychiatric	120 (53.3)	54 (53.5)	33 (56.9)	7 (53.8)
	Hepatic	157 (69.8)	83 (82.2)	39 (67.2)	9 (69.2)
	Other	90 (40.0)	40 (39.6)	27 (46.6)	8 (61.5)
	Asymptomatic	23 (10.2)	4 (4.0)	6 (10.3)	2 (15.4)
Follow-up since diagnosis, mean, months		46.8 (20.1)	43.2 (16.5)	51.6 (22.1)	40.2 (18.4)

Values are n (%) unless otherwise specified. Percentages are of the total number of patients receiving that specific therapy.

CCI, Charlson Comorbidity Index; Q, quartile; SD, standard deviation; WD, Wilson disease.

### Treatment patterns

**Table 3** details patient treatment characteristics and treatment duration by line of therapy (n=222). The median (Q1–Q3) time from WD diagnosis to first line of therapy treatment initiation was 0.3 (0.0–1.0) months; median (Q1–Q3) first line of therapy treatment duration was 34.6 (25.4–49.0) months overall. **Supplementary Table 1** presents the mean doses of index monotherapy by dosing frequency.

**Table 3:** Treatment Characteristics by Line of Therapy.

Variable	All (N=222)^a^	Penicillamine (n=101)	Trientine (n=58)	Penicillamine + trientine (n=3)	Zinc (n=13)	Chelator + zinc (n=47)
Time from diagnosis to initiation of first line of therapy, months						
Mean (SD)	1.6 (4.3)	2.6 (5.8)	0.6 (2.2)	0.5 (0.4)	0.4 (0.5)	0.8 (2.4)
Median (Q1–Q3)	0.3 (0.0–1.0)	0.6 (0.2–1.6)	0.1 (0.0–0.5)	0.5 (0.1–0.9)	0.2 (0.0–0.9)	0.3 (0.0–0.6)
Duration of first line of therapy among all patients, months						
Mean (SD)	37.6 (23.2)	31.1 (19.0)	41.6 (26.7)	34.6 (9.0)	27.4 (23.5)	49.4 (22.1)
Median (Q1–Q3)	34.6 (25.4–49.0)	33.0 (13.1–40.5)	35.4 (26.2–61.1)	35.3 (25.4–43.3)	30.8 (5.8–39.1)	43.5 (31.3–68.5)
Patients who remained on first line of therapy						
n, %	167 (75.2)	71 (70.3)	44 (75.9)	1 (33.3)	8 (61.5)	43 (91.5)
Duration of first line of therapy among patients who remained on first line of therapy, months						
Mean (SD)	45.6 (19.4)	39.2 (14.0)	51.5 (22.4)	43.32 (NE)	41.0 (19.7)	51.0 (21.0)
Median (Q1–Q3)	37.9 (32.7–58.8)	34.6 (31.7–43.6)	44.4 (33.9–69.4)	43.3 (43.3–43.3)	35.9 (31.4–41.0)	46.3 (34.5–68.5)
Patients with a subsequent line of therapy						
n, %	30 (13.5)	20 (19.8)	8 (13.8)	0 (0.0)	2 (15.4)	0 (0.0)
Duration of first line of therapy among patients with a subsequent line of therapy, months						
Mean (SD)	11.6 (11.7)	10.3 (12.4)	16.4 (10.0)	0.0 (0.0)	4.5 (1.7)	0.0 (0.0)
Median (Q1–Q3)	8.1 (5.8–11.5)	7.4 (4.8–10.1)	11.7 (8.9–24.0)	0.0 (0.0–0.0)	4.5 (3.3–5.8)	0.0 (0.0–0.0)
Time from end of first line of therapy to start of second line of therapy, months						
Mean (SD)	0.7 (1.1)	0.9 (1.3)	0.4 (0.5)	0.0 (0.0)	0.4 (0.4)	0.0 (0.0)
Median (Q1–Q3)	0.2 (0.0–1.1)	0.3 (0.0–1.7)	0.0 (0.0–0.8)	0.0 (0.0–0.0)	0.4 (0.1–0.8)	0.0 (0.0–0.0)
Patients discontinuing first line of therapy without subsequent line of therapy						
n, %	25 (11.3)	10 (9.9)	6 (10.3)	2 (66.7)	3 (23.1)	4 (8.5)
Duration of first line of therapy among patients who discontinued treatment, months						
Mean (SD)	15.3 (20.1)	15.8 (21.3)	3.1 (6.7)	30.3 (7.0)	6.4 (6.4)	32.0 (29.0)
Median (Q1–Q3)	12.0 (0.6–24.0)	7.4 (1.0–24.0)	0.3 (0.3–0.6)	30.3 (25.4–35.3)	5.8 (0.3–13.1)	21.1 (12.2–51.8)

a Three patients were not treated with a pharmaceutical agent of interest and were therefore excluded from this analysis.

NE, not estimable; SD, standard deviation.

After initiating first line of therapy, most patients (167/222 [75.2%]) remained on first line of therapy for the study duration, 30/222 (13.5%) switched to second line of therapy, and 25/222 (11.3%) discontinued first line of therapy without transitioning to a subsequent line of therapy. The median (Q1–Q3) treatment duration was 37.9 (32.7–58.8) months for patients who remained on first line of therapy, 8.1 (5.8–11.5) months for patients who switched to second line of therapy, and 12.0 (0.6–24.0) months for patients who discontinued first line of therapy without a subsequent line of therapy. Of the patients who initiated second line of therapy, 23/30 (76.7%) remained on second line of therapy, 5/30 (16.7%) switched to third line of therapy, and 2/30 (6.7%) discontinued second line of therapy without subsequent therapy.

**Figure 1:**
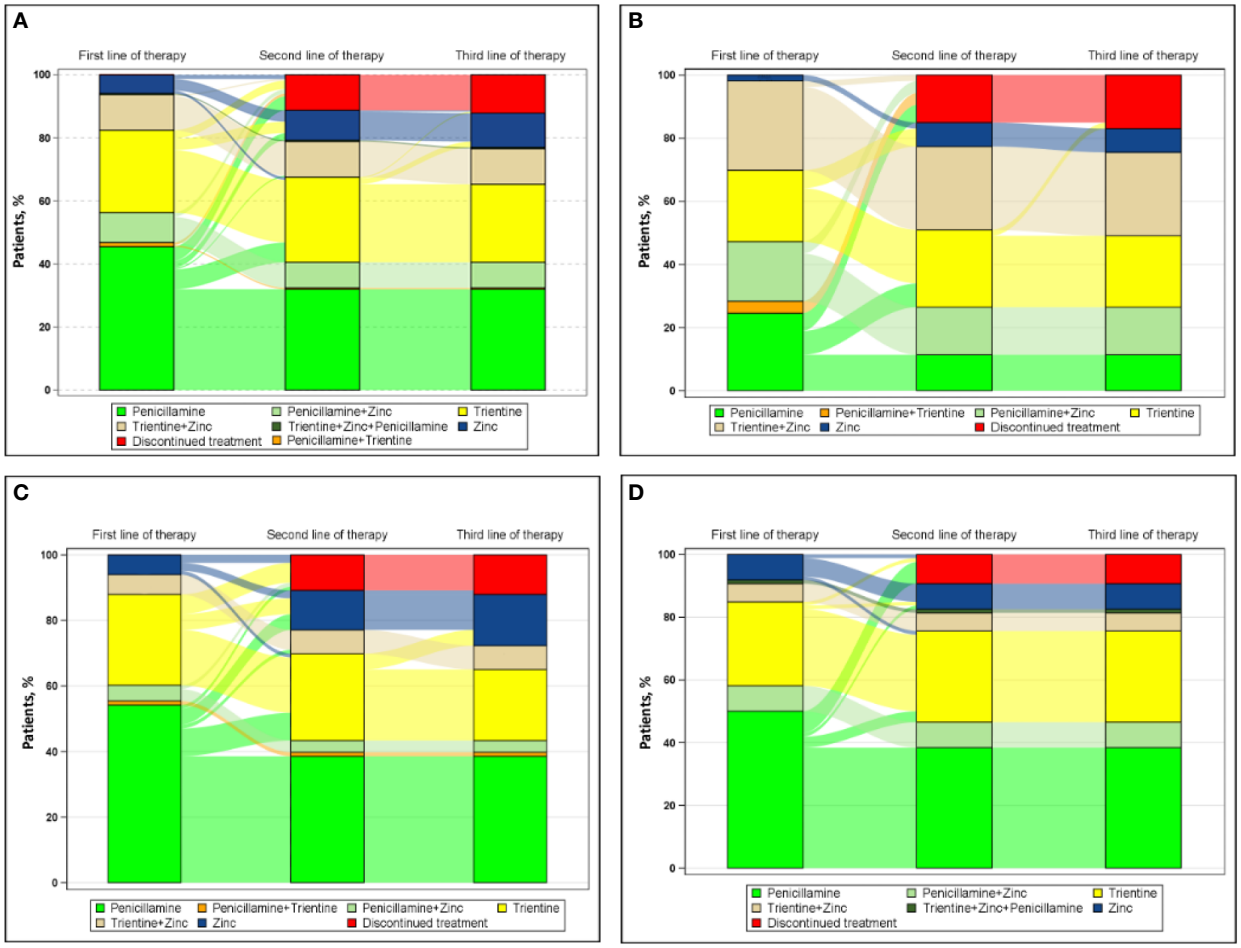
Sankey diagram of treatment patterns **(A)** overall (n=222) and among those treated by **(B)** neurologists (n=53), **(C)** hepatologists (n=83), and **(D)** gastroenterologists (n=86).

**Figure 1** shows the progression from first line of therapy through third line of therapy for all patients (**Figure 1A** [n=222]). The most common first line of therapy for all patients was penicillamine monotherapy (101/222 [45.5%]), followed by trientine monotherapy (58/222 [26.1%]) and chelator/zinc combination therapy (47/222 [21.2%]: penicillamine/zinc, 21/222 [9.5%]; trientine/zinc, 25/222 [11.3%]; penicillamine/trientine/zinc, 1/222 [0.5%]). The most common second line of therapy for all patients was trientine monotherapy (16/30 [53.3%]), followed by zinc monotherapy (14/30 [46.7%]), and the most common third line of therapy for all patients was zinc monotherapy (5/5 [100.0%]).

**Figure 1** also shows the progression from first line of therapy through third line of therapy for patients treated by physicians with different specialties, including neurologists (**Figure 1B** [n=53]), hepatologists (**Figure 1C** [n=83]), and gastroenterologists (**Figure 1D** [n=86]). For patients treated by neurologists, the most common first, second, and third line of therapy, respectively, was combination chelator/zinc therapy (25/53 [47.2%]), trientine monotherapy (4/7 [57.1%]), and zinc monotherapy (1/1 [100.0%]). For patients treated by hepatologists, the most common first, second, and third line of therapy, respectively, was penicillamine monotherapy (45/83 [54.2%]), both trientine (8/17 [47.1%]) and zinc monotherapy (8/17 [47.1%]), and zinc monotherapy (4/4 [100%]). For patients treated by gastroenterologists, the most common first and second line of therapy, respectively, was penicillamine monotherapy (43/86 [50.0%]) and trientine monotherapy (4/6 [66.7%]); no patient treated by a gastroenterologist received a third line of therapy during this study.

**Figure 2:**
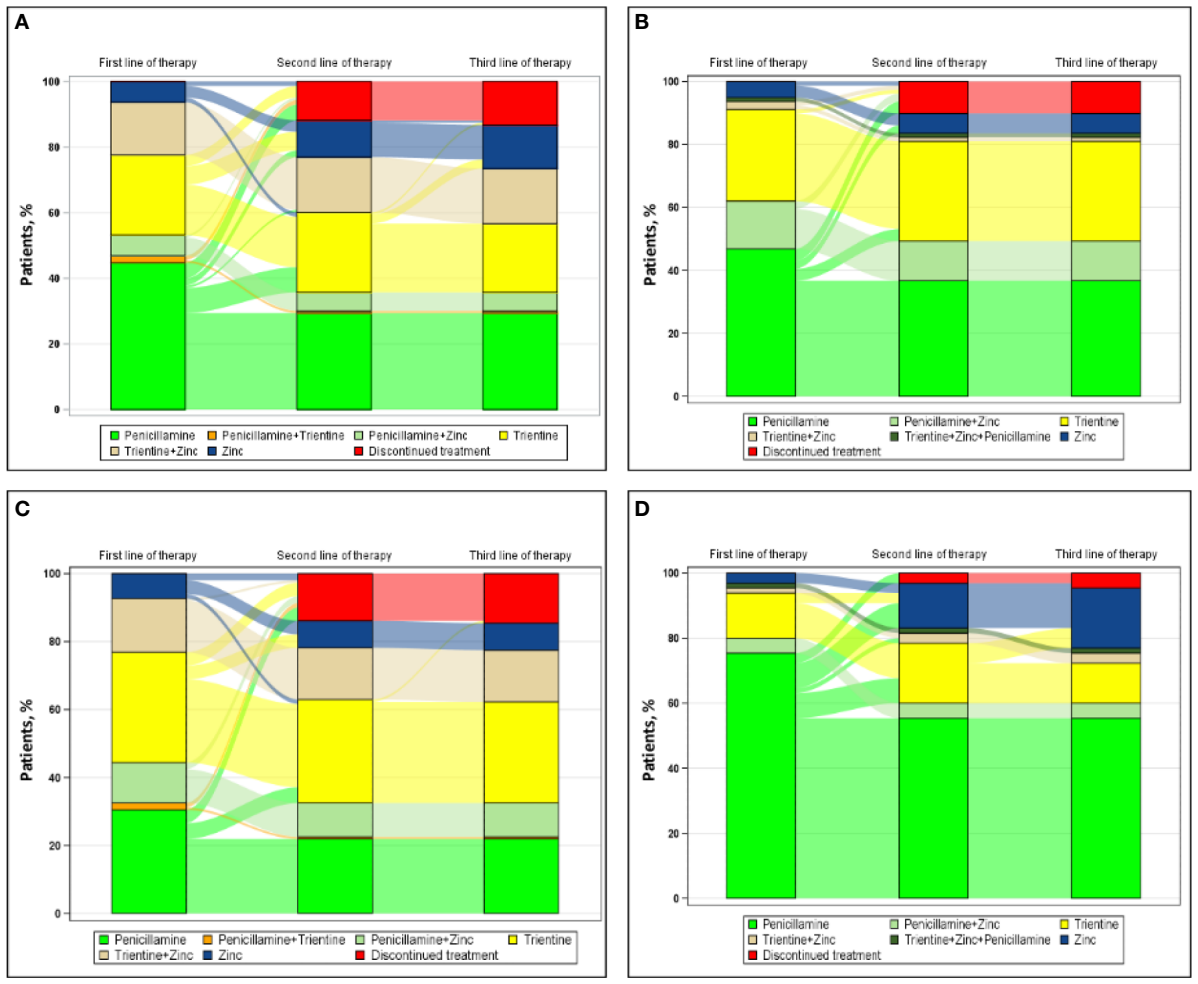
Sankey diagram of treatment patterns among those treated in **(A)** academic settings (n=143), **(B)** nonacademic settings (n=79), **(C)** urban regions (n=151), and **(D)** suburban regions (n=65).

**Figure 2** shows the progression from first line of therapy through third line of therapy for patients treated in academic (**Figure 2A** [n=143]) or nonacademic (**Figure 2B** [n=79]) settings. For patients treated in academic settings, the most common first, second, and third line of therapy, respectively, were penicillamine monotherapy (64/143 [44.8%]), trientine monotherapy (13/25 [52.0%]), and zinc monotherapy (5/5 [100.0%]). For patients treated in nonacademic settings, the most common first line of therapy and second line of therapy, respectively, were penicillamine monotherapy (37/79 [46.8%]) and trientine monotherapy (3/5 [60.0%]); no patients treated in nonacademic settings received a third line of therapy during the study.

**Figure 2** also shows the progression from first line of therapy through third line of therapy for patients treated in urban (**Figure 2C** [n=151]) or suburban (**Figure 2D** [n=65]) regions. Rural regions were omitted from this analysis because of insufficient sample size (n=6). In urban centers, the most common first, second, and third line of therapy, respectively, were trientine monotherapy (49/151 [32.5%]), trientine monotherapy (9/15 [60.0%]), and zinc monotherapy (1/1 [100.0%]). In suburban regions, the most common first, second, and third line of therapy, respectively, were penicillamine monotherapy (49/65 [75.4%]), zinc monotherapy (7/13 [53.8%]), and zinc monotherapy (4/4 [100.0%]).

**Figure 3:**
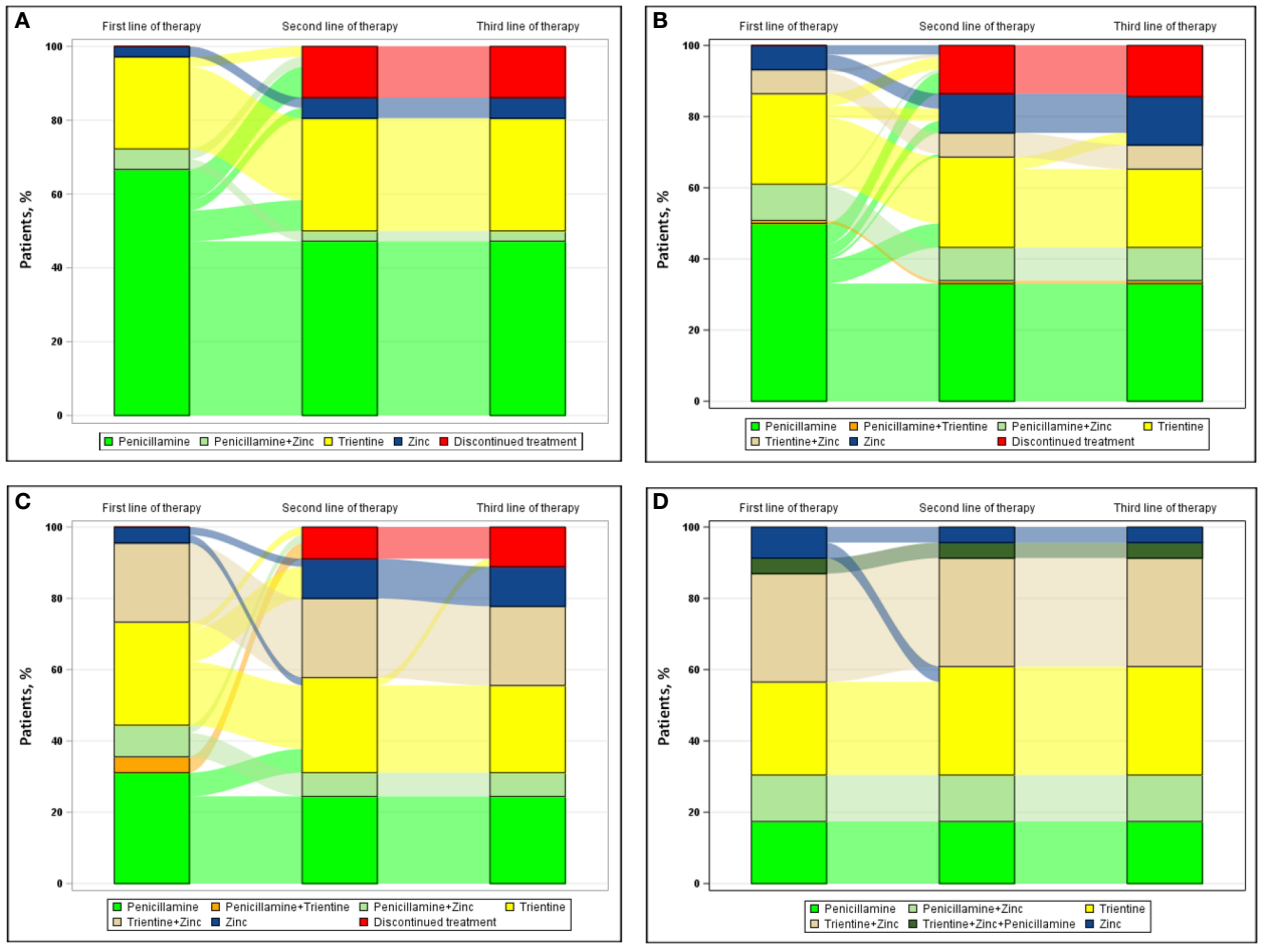
Sankey diagram of treatment patterns among those with **(A)** hepatic presentation (n=36), **(B)** neurologic/psychiatric and hepatic presentation (n=118), **(C)** neurologic/psychiatric presentation (n=45), and **(D)** asymptomatic presentation (n=23).

**Figure 3** shows the progression from first line of therapy through third line of therapy according to types of clinical presentations at diagnosis, including hepatic presentation (**Figure 3A** [n=36]), combined neurologic/psychiatric and hepatic presentation (**Figure 3B** [n=118]), neurologic/psychiatric presentation (**Figure 3C** [n=45]), and asymptomatic presentation (**Figure 3D** [n=23]). For patients with hepatic presentation, the most common first and second line of therapy, respectively, were penicillamine monotherapy (24/36 [66.7%]) and trientine monotherapy (3/4 [75.0%]); no patient with hepatic presentation at diagnosis received a third line of therapy during the study. For patients with combined neurologic/psychiatric and hepatic presentation, the most common first, second, and third line of therapy, respectively, were penicillamine monotherapy (59/118 [50.0%]), either trientine (8/17 [47.1%]) or zinc monotherapy (8/17 [47.1%]), and zinc monotherapy (4/4 [100.0%]). For patients with neurologic/psychiatric presentation, the most common first line of therapy was either penicillamine monotherapy (14/45 [31.1%]) or chelator/zinc combination therapy (14/45 [31.1%]), the most common second line of therapy was either trientine monotherapy (4/8 [50.0%]) or zinc monotherapy (4/8 [50.0%]), and the most common third line of therapy was zinc monotherapy (1/1 [100%]). For asymptomatic patients, the most common first line of therapy and second line of therapy, respectively, were chelator/zinc combination therapy (11/23 [47.8%]) and trientine monotherapy (1/1 [100.0%]); no patient with an asymptomatic presentation at diagnosis received a third line of therapy.

### Rationale for treatment selection and discontinuation

**Figure 4:**
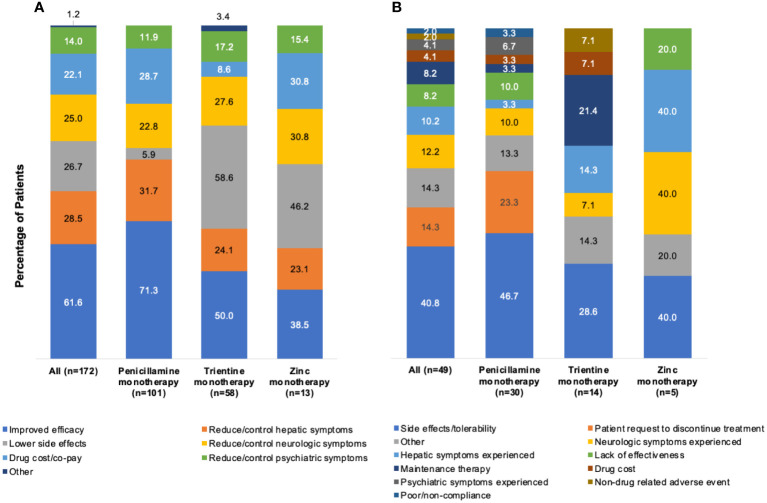
Rationales^a^ for **(A)** selection of index monotherapy and **(B)** discontinuation of index monotherapy. ^a^More than one rationale could be abstracted from each patient chart.

**Figures 4A, B** show physician rationales for selection of index monotherapy (n=172) and discontinuation (49/172 [28.5%]). Overall, the primary rationale for index monotherapy selection was perceived comparative efficacy (106/172 [61.6%]). Perceived efficacy was also the primary rationale for selecting index penicillamine monotherapy (72/101 [71.3%]). Lower side effects were the primary rationale for selecting index trientine monotherapy (34/58 [58.6%]) or zinc monotherapy (6/13 [46.2%]). Overall, the primary rationales for discontinuation of index monotherapy were side effects and tolerability (20/49 [40.8%]), including 14/30 (46.7%) for index penicillamine monotherapy and 4/14 (28.6%) for trientine monotherapy. Three factors were cited equally as primary rationales for discontinuing index zinc monotherapy: side effects/tolerability, hepatic symptoms, and neurologic symptoms (all 2/5 [40.0%]).

**Figure 5:**
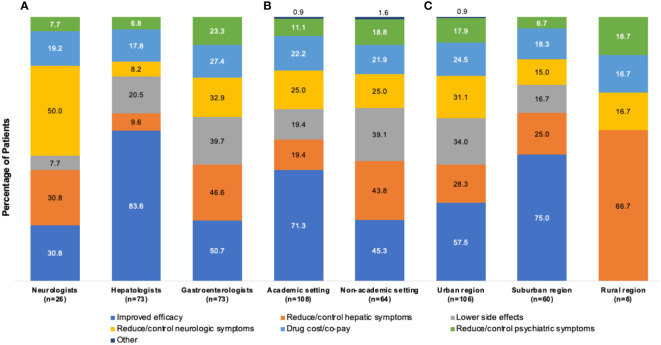
Rationales^a^ for selection of index monotherapy by **(A)** physician type, **(B)** practice setting, and **(C)** geographic location. ^a^More than one rationale could be abstracted from each patient chart.

**Figure 5A** shows the reported rationale for index monotherapy selection by physician specialty. The primary rationale of neurologists (n=26) for index monotherapy selection was efficacy in the reduction/control of neurologic symptoms (13/26 [50.0%]), followed by improved efficacy (8/26 [30.8%]) and reduction/control of hepatic symptoms (8/26 [30.8%]). For patients treated by hepatologists (n=73), the primary rationale was improved efficacy (61/73 [83.6%]), followed by lower side effects (15/73 [20.5%]) and drug cost/co-pay (13/73 [17.8%]). For patients treated by gastroenterologists (n=73), the primary rationale was improved efficacy (37/73 [50.7%]), followed by reduction/control of hepatic symptoms (34/73 [46.6%]) and lower side effects (29/73 [39.7%]).

**Figure 5B** shows the rationale for index monotherapy selection according to academic or nonacademic practice settings. For patients treated in an academic setting (n=108), the primary rationale for index monotherapy selection was comparative efficacy (77/108 [71.3%]), followed by reduction/control of neurologic symptoms (27/108 [25.0%]) and drug cost/co-pay (24/108 [22.2%]). For patients treated in a nonacademic setting (n=64), the primary rationale was comparative efficacy (29/64 [45.3%]), followed by reduction/control of hepatic symptoms (28/64 [43.8%]) and lower side effects (25/64 [39.1%]).

**Figure 5C** shows the rationale for index monotherapy selection according to geographic region. For patients treated in an urban location (n=106), the primary rationale for index monotherapy selection was comparative efficacy (61/106 [57.5%]), lower side effects (36/106 [34.0%]), and reduction/control of neurologic symptoms (33/106 [31.1%]). For patients treated in a suburban location (n=60), the primary rationale was comparative efficacy (45/60 [75.0%]), followed by reduction/control of hepatic symptoms (15/60 [25.0%]) and drug cost/co-pay (11/60 [18.3%]).

## Discussion

This retrospective, observational study provides the first real-world analysis of patients with WD in the United States to answer 3 key questions regarding clinical presentations; choices of first and subsequent line therapies by physicians of different specialties, practice settings, and geographic practice locations; and physician use of published WD guidelines. On average, patients experienced their first symptoms at age 23.2 years, were diagnosed over 1 year later at age 24.7 years, and had received treatment for approximately 3 years. Patients were mostly male, White, commercially insured, and without a known family history of WD. The most common presentation at WD diagnosis was combined neurologic/psychiatric and hepatic disease (52.9%). Only 40.9% of treating physicians used recommendations in published WD guidelines. This is in accordance with the fact that only 57.7% of patients were advised to restrict dietary copper.

The treatment patterns observed in this study indicate a lack of consensus regarding initial treatment of patients with WD among neurologists, gastroenterologists, and hepatologists, and a continued reliance on traditional penicillamine monotherapy as a first of therapy mainstay, primarily for the perception of improved efficacy (20). Specifically, 45.5% of patients received penicillamine monotherapy as first line of therapy, while 47.3% were treated with trientine monotherapy or chelator/zinc combination therapy as first line of therapy. As anticipated, penicillamine often caused adverse events and tolerability issues, which necessitated switching of therapy in up to 36% of cases (3, 20, 30–32) Indeed, in the current study, a lower risk of side effects was the primary rationale of physicians for initiating first line of therapy monotherapy with trientine (56.6%) or zinc (46.2%). However, the perception that penicillamine had greater efficacy than trientine or zinc was the primary rationale for first line of therapy penicillamine monotherapy (71.3%). Clinical guidance recommendations that conflict with the current US prescribing information for trientine, as well as the high cost of trientine may have also contributed to the choice of first line of therapy. Clinical guidelines recommend first-line chelator therapy with either penicillamine or trientine (3, 22, 27), but use of trientine as first line of therapy monotherapy is still considered off-label (33). US prescribing information authorizes trientine use only when “continued treatment with penicillamine is no longer possible because of intolerable or life endangering side effects.” (33) High-quality, prospective, randomized trials demonstrating the comparative treatment efficacy and safety of first-line penicillamine, trientine, and zinc are needed to refine treatment decisions, clarify and extend clinical guideline recommendations, and update approved indications for currently available therapies.

Choice of first line of therapy may also reflect physician experience, drug availability, cost, and insurance coverage (34). In suburban regions, penicillamine was first line of therapy for the vast majority (75.4%) of patients, a numerically higher proportion than any other physician subgroup (the next highest was among gastroenterologists [50.0%]). The primary rationale was improved efficacy (75.0%); this suggests that physicians practicing in suburban centers may not have sufficient experience with alternative first line of therapy choices. According to the AASLD and EASL guidances on WD, treatment targets for therapies include urinary copper excretion and symptomatic control (26, 27). With respect to drug availability, drug cost/co-pay was a consideration in the selection of first line of therapy across the board for approximately 20% of patients who were treated by neurologists or hepatologists, in academic or nonacademic settings, or suburban settings and for approximately 25% of patients treated by gastroenterologists and in urban settings. The unfortunate rise in the cost of trientine imposed by its manufacturer led to unaffordability owing to high co-pays (35, 36). It also led some insurers to refuse use of trientine unless a patient has a proven intolerance to penicillamine (33, 37–39). Therefore, physicians may refrain from prescribing trientine to avoid dealing with insurance denials.

With respect to combination therapy as first line of therapy, the current AASLD guidance recommends chelator/zinc combination therapy for more severe disease (e.g., decompensated cirrhosis) but notes that combination therapy remains investigational (26). Indeed, no prospective randomized trials have been conducted to evaluate the efficacy and safety of chelator or zinc monotherapies versus combination chelator/zinc therapy (1). Despite the absence of an evidence basis for use of combination chelator/zinc therapy, combination therapy with trientine/zinc as first line of therapy was particularly common among US patients treated by neurologists, among patients with neurologic/psychiatric or asymptomatic presentation, and among patients treated in either an academic or urban setting. This may be a result of sufficient clinical experience to consider this approach valid. More research is needed on the comparative efficacy and safety of monotherapy versus combination therapy for WD.

This study also identified an unmet need for an evidence-based treatment approach for patients with neurologic WD. Despite the risks of progressing or worsening neurologic WD symptoms with penicillamine, owing to the rapidity of copper extraction from the nervous system (40), nearly one half of first line of therapy regimens prescribed by neurologists in the current study used penicillamine monotherapy or in combination with zinc. Non-neurologists prescribed penicillamine to a similar percentage of patients with neurologic/psychiatric disease presentations. A systematic review and meta-analysis of 16 cohort studies published in 2022 compared the efficacy and safety of penicillamine or zinc for symptomatic WD. Among symptomatic patients with WD (N=1033), penicillamine increased the relative risk of neurological worsening to 1.96 (95% CI: 1.31–2.93; *P*=0.001) compared with zinc (41). A 2009 systematic review of the clinical efficacy of WD treatments, which included 1 randomized controlled trial and 12 observational studies, reported deterioration of neurologic signs or symptoms after initiation of therapy in 6/107 (5.6%) of patients treated with penicillamine, but only 1/127 (0.8%) of patients treated with zinc (40). The EASL clinical practice guidelines note that initial treatment with zinc may be better tolerated than penicillamine for patients with neurologic disease presentation (22). AASLD 2022 guidelines do not provide specific recommendations regarding choice of therapy. It is unclear if any of the three major WD clinical practice guidelines are widely used by neurologists in the US.

Finally, in the current study, 11.3% of patients discontinued their first line of therapy without transitioning to a subsequent line of therapy, most often because of side effects/tolerability. This is concerning, as patients with WD who permanently discontinue treatment are at increased risk of organ failure and earlier death (1, 12, 42). Discontinuation of treatment because of side effects has been reported, and a recent single-center retrospective audit of data from 112 patients treated with penicillamine between 2006 and 2020 showed that 16/112 (14.3%) permanently discontinued treatment because of severe adverse effects (43). Similarly, a 2009 systematic review noted a 12.5% (28/224 patients) rate of permanent discontinuation of penicillamine because of severe side effects (40). Discontinuation without prescription of alternative therapy must be discouraged and presents an important unmet need for education among physicians treating patients with WD and among patients with WD.

### Strengths and limitations

The strengths of this study in the United States include its large sample size and use of data abstracted directly from patient charts. This approach permitted analysis of initial and subsequent treatments, which would have been challenging with other data sources, such as administrative claims. Furthermore, this real-world evidence–based study increases the inclusion of patients who may otherwise have been excluded from clinical trials because of age, comorbidities, or other factors (44).

However, this study also has limitations. By focusing on a limited number of clinicians with differing medical specialties, the results may not be generalizable to all US patients with WD. Of note, physicians reporting that their therapeutic decisions were based on their views of comparative efficacy among treatment choices were not queried about the evidence basis for their choice. Furthermore, this study did not capture data on patient dietary restrictions or its relation to the selection of therapy. Additionally, since the average follow-up was only four years from diagnosis, patients were relatively early in their treatment journey, which is lifelong. Finally, small subgroup sample sizes (e.g., patients initiating treatment with zinc monotherapy) warrant caution when interpreting results. Health inequities in the US that contribute to racial disparities in access to healthcare may have contributed to the overrepresentation of white patients and might not be representative.

## Conclusion

This study provides the first descriptive real-world data in the US about treatment of patients with WD. Approximately half of the physicians in this study do not follow published clinical practice guidance with respect to dietary copper restriction or recommendations for first-line therapy. While penicillamine was the most common first-line therapy overall, treatment patterns varied widely by physician specialty, clinical presentation, practice setting, and geographic location. The most prominent first-line therapies among asymptomatic patients and those with neurologic disease treated by neurologists were trientine monotherapy and combination chelator/zinc therapy. Evidence-based treatment is needed for patients with this rare and progressive disease to improved clinical outcomes. Development of new therapies, supported by high-quality randomized, controlled clinical trials, research on the comparative effectiveness of available treatments, and promotion of clinical guidelines to practicing physicians are all needed to achieve better outcomes for patients with WD.

## Data availability statement

The data analyzed in this study is subject to the following licenses/restrictions: Alexion, AstraZeneca Rare Disease will consider requests for disclosure of clinical study participant-level data provided that participant privacy is assured through methods like data de-identification, pseudonymization, or anonymization (as required by applicable law), and if such disclosure was included in the relevant study informed consent form or similar documentation. Qualified academic investigators may request participant-level clinical data and supporting documents (statistical analysis plan and protocol) pertaining to Alexion-sponsored studies. Further details regarding data availability and instructions for requesting information are available in the Alexion Clinical Trials Disclosure and Transparency Policy at https://alexion.com/our-research/research-and-development. Requests to access these datasets should be directed to Link to Data Request Form (https://alexion.com/contact-alexion/medical-information).

## Ethics statement

The studies involving humans were approved by New England Institutional Review Board (Needham, MA). The studies were conducted in accordance with the local legislation and institutional requirements. Written informed consent for participation was not required from the participants or the participants’ legal guardians/next of kin in accordance with the national legislation and institutional requirements.

## Author contributions

VM: Investigation, Writing – review & editing. NK: Data curation, Formal analysis, Methodology, Writing – review & editing. JS: Conceptualization, Investigation, Supervision, Writing – original draft, Writing – review & editing. MK: Investigation, Writing – review & editing. JV: Investigation, Writing – review & editing.
