# Supplementary material for: Treatment patterns in a real-world cohort of patients with Wilson disease in the United States
Source: Front Gastroenterol (Lausanne). 2024 May 24;3:1363130. doi: 10.3389/fgstr.2024.1363130 (PMC12952460; doi:10.3389/fgstr.2024.1363130)
Supplement: Supplementary file 1 [file Table_1.docx]

**Treatment Patterns in a Real-World Cohort of Patients With Wilson Disease in the United States** Medici et al.

**Supplemental Table 1.** Initial Mean (SD) Dose of Index Monotherapy by Dosing Frequency

| **Variable** | **Statistic/Category** |  |
| --- | --- | --- |
| Penicillamine monotherapy, mg | All | n=101  338.6 (125.9) |
|  | QD | n=11  363.6 (130.6) |
|  | BID | n=17  350.0 (157.1) |
|  | TID | n=37  412.2 (121.0) |
|  | QID | n=36  250.0 (0.0) |
| Trientine monotherapy, mg | All | n=58  424.1 (212.2) |
|  | QD | n=1  250.0 (NE) |
|  | BID | n=28  530.4 (240.5) |
|  | TID | n=5  343.7 (123.6) |
|  | QID | n=5  250.0 (0.0) |
| Zinc monotherapy, mg | All | n=13  44.2 (11.0) |
|  | QD | n=2  37.5 (17.7) |
|  | BID | n=1  50.0 (NE) |
|  | TID | n=10  45.0 (10.5) |
|  | QID | n=0  0 |

NE, not estimable.
